# A rare case of penile schwannomatosis presenting with painful nocturnal penile tumescence

**DOI:** 10.1186/s12610-022-00154-y

**Published:** 2022-03-21

**Authors:** Chan Ming Tow, Jonathan Tang, Chan Ming Chun, Joe Lee King Chien

**Affiliations:** 1 Department of Urology, National University Hospital, 5 Lower Kent Ridge Rd, Singapore, 119074 Singapore; 2 Department of Pathology, National University Hospital, 5 Lower Kent Ridge Rd, Singapore, 119074 Singapore; 3 Department of Urology, Tan Tock Seng Hospital, 11 Jln Tan Tock Seng, Singapore, 308433 Singapore

**Keywords:** Penile, Schwannoma, Painful tumescence, Sexual dysfunction, Pénis, schwannome, tumescence douloureuse, dysfonction sexuelle

## Abstract

**Background:**

Penile schwannoma is a rare tumor. They commonly present as an asymptomatic, painless and slow growing mass. Other presentations include sexual dysfunction, most commonly dyspareunia, followed by erectile dysfunction, abnormal penile curvature or pain with ejaculation.

**Case presentation:**

A 26-year-old male presented atypically with painful nocturnal penile tumescence, along with multiple nodules over the dorsal penis. Excision of multiple penile tumors under general anaesthesia was performed and histopathologic examination revealed benign schwannoma.

**Conclusion:**

Our hypothesis is that the schwannoma lies along the axis of the dorsal penile nerve, and compression of this nerve occurs during his erection causing pain. However, there are limited presentations of painful erections in penile schwannomas, and we hope that future studies can help confirm this theory.

## Abstraite

**Contexte:**

Le schwannome pénien est. une tumeur rare. Il se présente généralement comme une masse asymptomatique, indolore et à croissance lente. D’autres présentations incluent la dysfonction sexuelle, le plus souvent la dyspareunie, suivie de la dysfonction érectile, de la courbure anormale du pénis ou de la douleur à l’éjaculation.

**Présentation du cas:**

Un homme de 26 ans s’est. présenté de façon atypique avec une tumescence pénienne nocturne douloureuse, ainsi que de multiples nodules sur la face dorsale du pénis. L’excision de plusieurs tumeurs du pénis a été réalisée sous anesthésie générale et un examen histopathologique a révélé un schwannome bénin.

**Conclusion:**

Notre hypothèse est. que le schwannome se trouve localisé le long de l’axe du nerf pénien dorsal, et que la compression de ce nerf se produit pendant l’érection, constituant la source des douleurs. Cependant, il existe peu de présentations d’érections douloureuses dans les schwannomes péniens, et nous espérons que de futures études pourront aider à confirmer cette théorie.

## Background

Schwannomas are a form of peripheral nerve tumors made up of neoplastic Schwann cells that typically occur as solitary, encapsulated masses. They can occur throughout the body, but more commonly arise on the head, neck, or flexor surfaces of limbs [1, 2]. The tumors are sporadically associated with genetic syndromes such as schwannomatosis and neurofibromatosis, or may be the result of therapeutic irradiation [3]. Schwannomas have a low annual incidence of 0.6 per 100,000 people [4]. These are rare and only 27 cases have been reported in literature since it was first described in 1968 [5]. Penile schwannomas are typically asymptomatic, painless and slow growing. Possible presentations include sexual dysfunction, most commonly dyspareunia, followed by erectile dysfunction, abnormal penile curvature, or pain with ejaculation. Our patient presented with painful nocturnal penile tumescence, which is not a well-known presentation of penile schwannomas. There is limited published literature on such cases and hence little is known about this condition.

## Case presentation

A 26-year-old man with a history of ankylosing spondylitis (Human Leukocyte Antigen B27 gene) and previous circumcision first presented with a one-year history of recurrent painful nocturnal erections. He had prior consultations with various urologists and did not respond to oral analgesia. The frequency of painful nocturnal erections increased from once per week, to thrice per week over the past year. Each episode of painful nocturnal tumescence lasted approximately 5 minutes and the patient was often awaken from sleep by the severe pain, which affected his sleep and quality of life. The patient mentioned that the nocturnal erections were stronger and more painful compared to when he was awake. There was no history of priapism, sexual transmitted disease, or genital trauma. There were no persons with known neurofibromatosis in his family. On examination, four lumps could be palpated over the dorsum of the stretched penis. Two were superficial nodules on the distal shaft, with one deep nodule each at the mid shaft and base of the penis. The nodules were 0.5 cm or less in diameter and firm in nature. The mid shaft nodule was tender on palpation and correlated with the site of painful nocturnal erections. The penis was otherwise unremarkable and there was neither penile curvature on erection nor any palpable lymph nodes in the femoral or inguinal areas. Nodules or café-au-lait spots were not present in the rest of the body.

Over a five-year follow-up, patient developed worsening symptoms with the painful erections occurring twice every night from one episode a week. Physical examination and interval ultrasound imaging demonstrated an increase in the number of nodules from four to five with further growth of the existing nodules. Most noticeably, the right intracavernosal nodule increased from 4 mm to 7 mm in diameter. The patient decided for surgical excision of multiple penile nodules.

## Investigations

Laboratory findings included normal blood cell counts, chemistries and urinalysis.

**Fig. 1:**
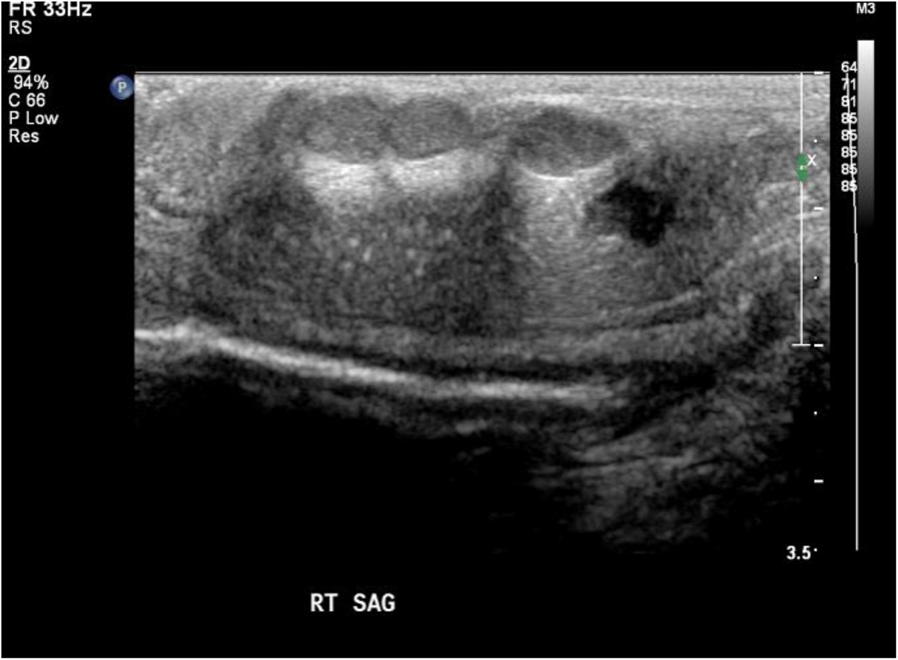
Ultrasound image (longitudinal view) of penile nodules. Ultrasound image (longitudinal view) of penile nodules demonstrates three well-circumscribed, hypoechoic nodules adjacent to the dorsal aspect of the corpus cavernosa

**Fig. 2:**
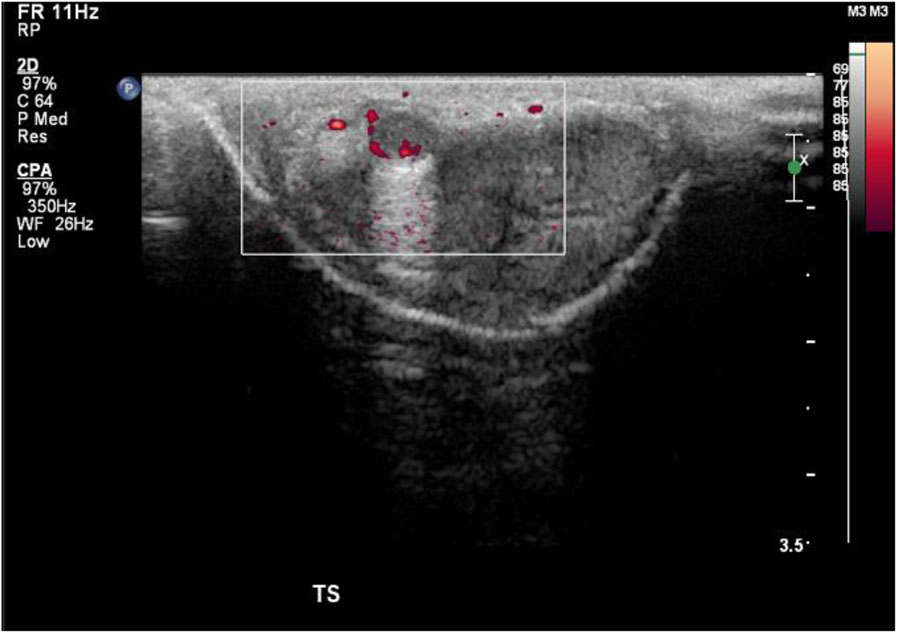
Ultrasound image (longitudinal view) of penile nodules with power doppler interrogation. Ultrasound image (longitudinal view) with power doppler interrogation reveals the presence of internal vascularity within the nodule

**Fig. 3:**
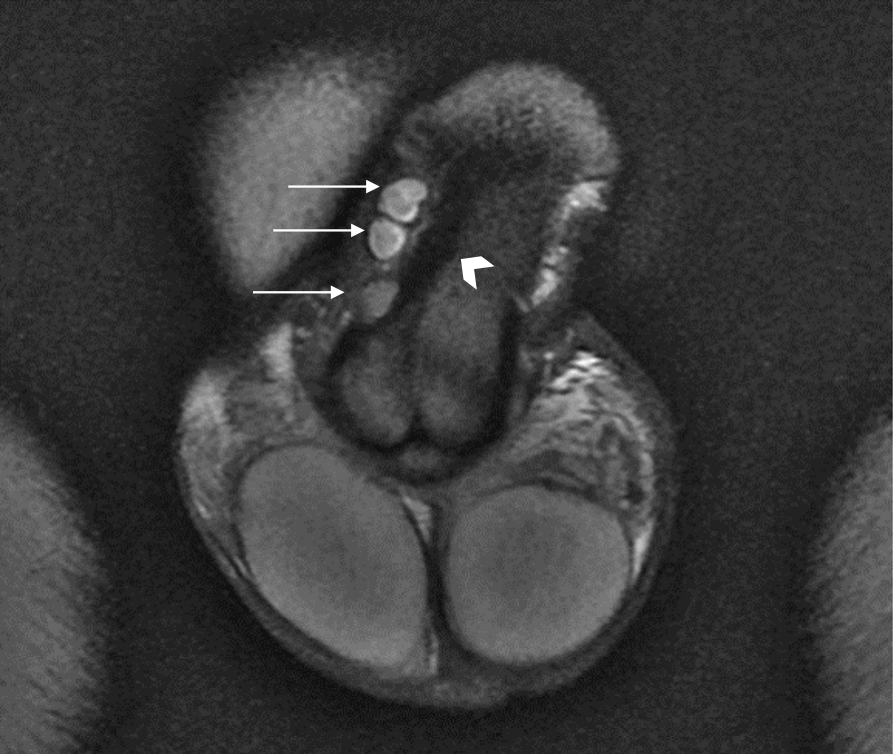
Coronal T2-weighted magnetic resonance image (MRI) of penile nodules. Coronal T2-weighted MR image shows three well-defined T2W hyperintense nodules (arrows) within the penis. These are superficial to the tunica albuginea (arrowhead)

**Fig. 4:**
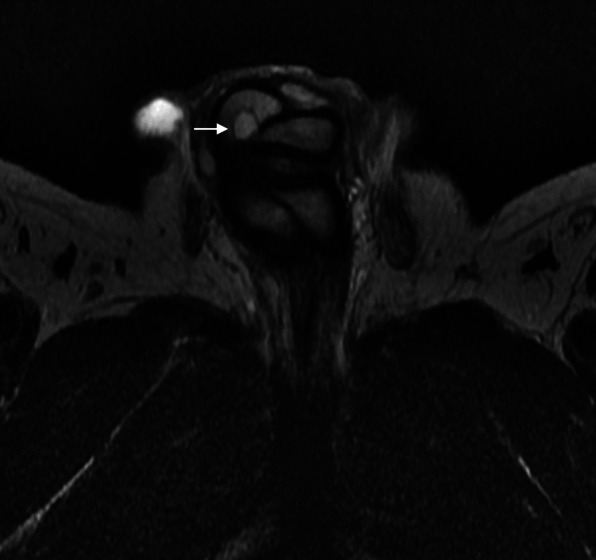
Axial T2-weighted MRI of penis and nodule. Axial T2-weighted MRI of hyperintense nodule (arrow) is seen within the right corpus cavernosa

**Fig. 5:**
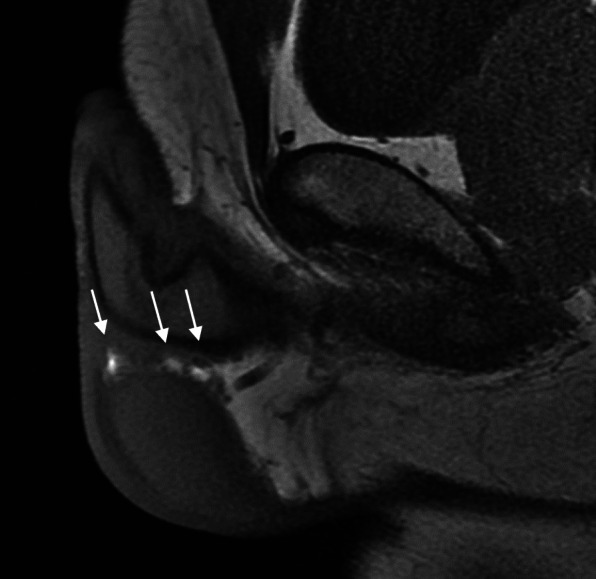
Sagittal T1-weighted post contrast MRI of penile nodules. Sagittal T1-weighted post contrast MRI of penile nodules displaying low signal intensity (arrows)

**Fig. 6:**
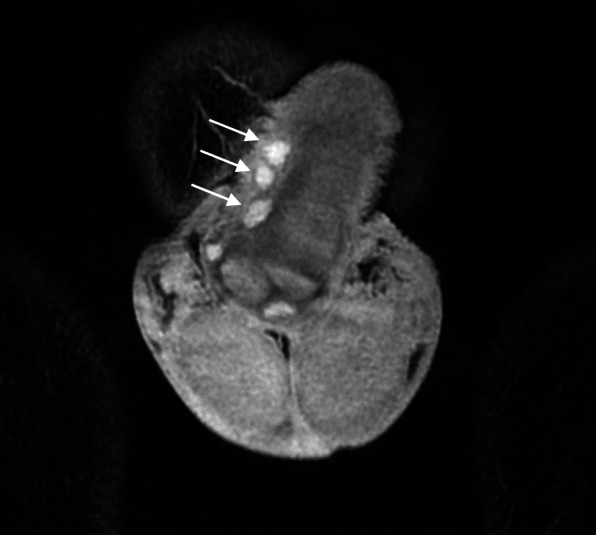
Coronal T1-weighted post contrast MRI of penile nodules. Coronal T1-weighted post contrast MRI showing penile nodules of low signal intensity with homogenous and avid post contrast enhancement (arrows)

Initial ultrasound penis showed multiple rounded heterogeneously echogenic nodules in the subcutaneous region of the dorsal penile shaft (Fig. 1). The nodules show minimal central and peripheral vascularity (Fig. 2). The patient initially declined surgical intervention and opted for annual ultrasound imaging. Magnetic resonance imaging (MRI) of the penis performed prior to surgery showed multiple enhancing sub-centimetre nodules in the penile shaft, most of which were superficial (Fig. 3,4). These nodules display low signal intensity with homogenous and avid post contrast enhancement (Figs. 5 and 6). There was also a nodule in the right corpus cavernosum (Fig. 4).

## Treatment

**Fig. 7:**
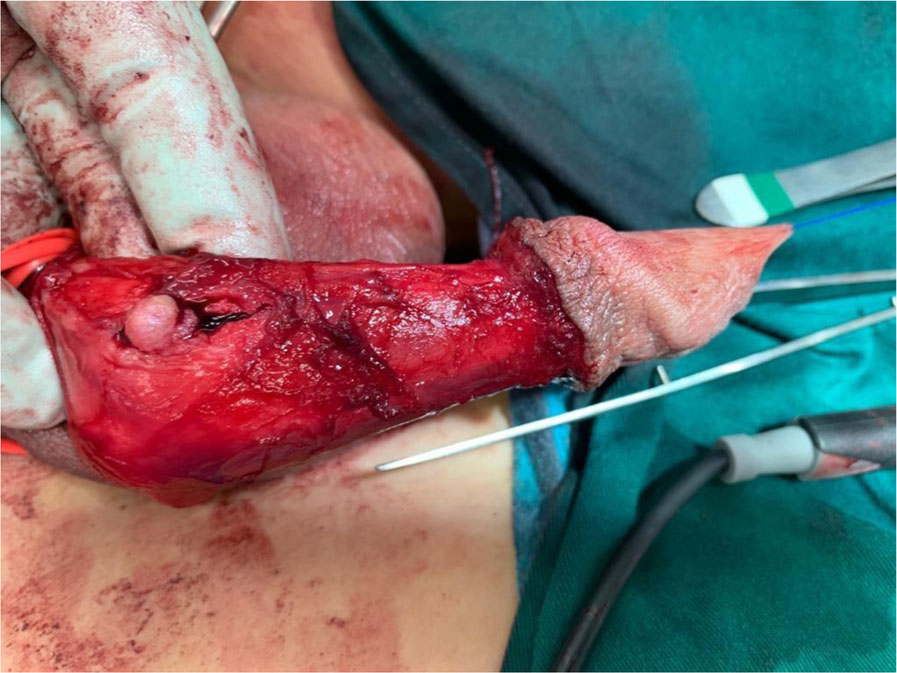
Intra-operative image of excision of penile nodule. Intra-operative image of excision of penile nodule where the penis has been degloved to its base and the layers dissected down to Buck’s fascia. A superficial tumor measuring 1 cm in diameter can be seen

Patient underwent excision of multiple penile tumors under general anaesthesia. A circumferential incision was made at the previous circumcision site. The penis was then degloved to its base and the layers dissected down to Buck’s fascia (Fig. 7). There were five superficial tumors adherent to the tunica albuginea (two at right distal shaft, two at midshaft, one at base of penis). A deep-seated tumor was located at the right corporal mid shaft. The tumors measured approximately 1–1.5 cm in diameter. The cut surface of the tumors were homogenously yellowish with noted feeding vessels. All tumors were excised and histology was sent from all locations.

## Outcome and follow-up

**Fig. 8:**
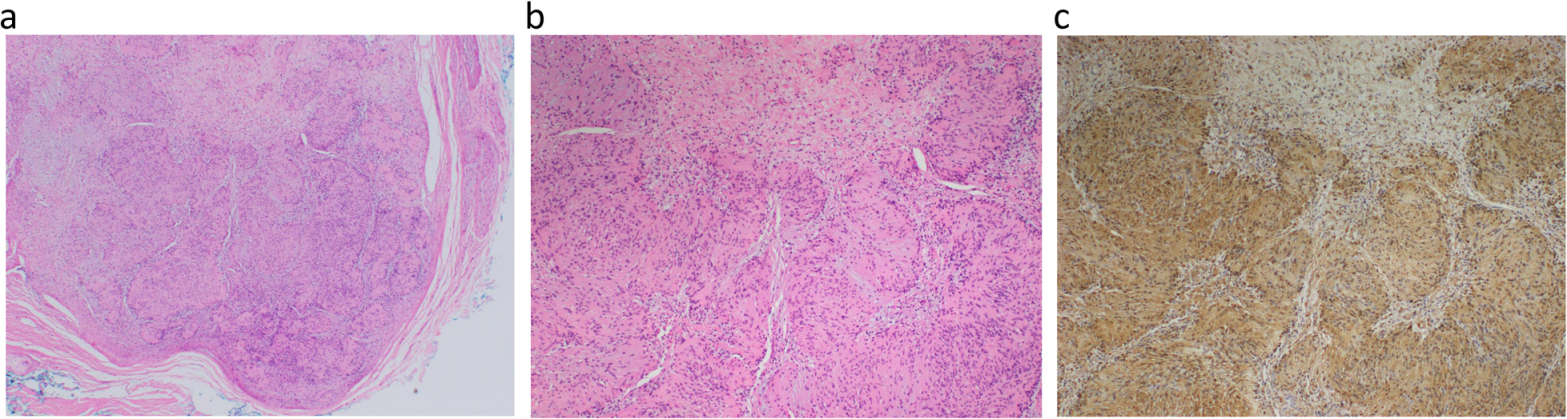
**a**, **b**, **c** Histologic section from excision biopsies. **a** Well-circumscribed and thinly encapsulated tumour nodule (Hematoxylin and Eosin stain, 20x magnification). **b** Cellular Antoni A areas with short fascicles of Schwann cells and less cellular Antoni B areas with myxoid stroma (Hematoxylin and Eosin stain, 200x magnification). **c** Diffuse and strong S100 expression in the tumour cells (S100 immunohistochemistry stain, 200x magnification)

The Hematoxylin and Eosin stains and immunohistochemical studies were performed in accordance with local laboratory protocols. All specimens show similar morphology. The circumscribed and thinly encapsulated nodules (Fig. 8a) were made up of Schwann cells arranged as a mixture of more cellular Antoni A and less cellular Antoni B areas. The more cellular Antoni A areas consists of Schwann cells arranged as short fascicles or parallel rows of nuclear pallisading (Verocay bodies). The less cellular Antoni B areas show a more loose myxoid stroma (Fig. 8b). The nodules are associated with thickened and oedematous nerve fibres, and occasional more plexiform Schwannian areas are seen involving the nerve fibres. No high-grade nuclear atypia, increased mitosis or tumour necrosis is seen. On immunostaining, the lesion shows diffuse staining with S-100 (Fig. 8c), which is indicative of Schwannoma.

In the follow up consultations over a year after surgery, there was no further nightly painful erections and patient was able to sleep well. On examination there is a small nodularity at mid shaft which is likely due to scar tissue formation. However, the patient did experience difficulty maintaining erection due to discomfort over the surgical site. This was managed well with Sildenafil 25 mg, with an improvement in International Index of Erectile Function [6] score from 8/25 to 19/25. Patient declined genetic testing as he was not keen on childbearing.

## Discussion

Schwannomas rarely present with penile pain. There have been postulations made regarding the correlation of symptoms to neuroanatomy. A literature review done by Huang et al. concluded that patients with penile root schwannomas are more prone to symptoms with discomfort or sexual dysfunction (4 of 6) compared with patients with penile shaft schwannoma (7 of 16) or glans schwannoma (2 of 7) [7]. Based on anatomy, penile schwannomas at the mid shaft or glans should originate from the dorsal nerve of the penis, which is the deepest division of the pudendal nerve. The pudendal nerve does pass through the penile root, but there is no clear branching or tracking of the nerve origin of the tumor [8]. Pain may occur in the region of the tumor and any nerve the tumor originates from, but pain may not be specific enough to discern the particular involved nerve. Neurologic deficits of sensory and motor function correspond to the nerve in which the tumor originates or which it is compressing, and as such will often be most useful in localizing the tumor [9, 10]. This patient presented with painful nocturnal erections corresponding to the mid shaft schwannoma. Our hypothesis is that the mid shaft schwannoma lies along the axis of the dorsal penile nerve and pain could arise when the nerve is compressed by the schwannoma during full erection. During nocturnal tumescence, the cavernosus arteries dilate, leading to engorgement of the corpora cavernosa and increase of intra-corporal pressure. This pushes the schwannoma towards the dorsal penile nerve leading to nerve irritation, compression and pain.

Penile schwannomas normally occur at the dorsal penile shaft. However, there have been documented cases where the tumor has infiltrated the glans and prepuce [11]. In such cases, we have to consider other possible diagnoses including benign soft-tissue lesions such as lipoma, fibroma, leiomyoma, Peyronie’s disease, injection-related fibrosis, and rarely malignant sarcomas. Clinical history taking and clinical examination are important, but imaging can aid in narrowing the differentials by locating the plane of the lesion and delineating the mass. Ultrasound examination can demonstrate hypoechoic lesions, and doppler ultrasound can detect hypervascularity. Computed tomography scan is rarely used, and mostly performed to exclude metastasis. Schwannomas demonstrate typical MRI features of T1 isointensity to hypointensity, T2 hyperintensity, and postcontrast enhancement. Heterogeneous signal intensity and postcontrast enhancement are suggestive of internal hemorrhage and myxoid/cystic changes [12]. Otherwise, excision biopsy of the tumor would be the gold standard for final diagnosis. Treatment of penile schwannomas is symptomatic, focused primarily on pain management. Complete surgical excision is the recommended treatment for penile schwannomas, with low recurrence rates [1, 2, 10]. This patient recovered well with no signs of recurrence 1 year postoperatively.

Schwannomas of the penis are usually benign, but four malignant variants have been reported in literature. No cases of benign penile schwannoma have been reported to be associated with hereditary diseases [14]. Schwannomatosis is the third major form of neurofibromatosis, and is characterized by a predisposition for schwannomas, in the absence of schwannomas on both vestibular nerves. Its diagnosis is based on a criterion [15]. Most patients present in adulthood with multiple schwannomas and pain, and approximately 20% of patients have a family history of schwannomas or schwannomatosis [16]. So far, there have been no confirmed causes of penile schwannoma with schwannomatosis.

There is no strong evidence about the correlation between schwannoma and erectile dysfunction [13]. This patient’s postoperative erectile dysfunction is likely due to pain surrounding the surgical wound site. Also, there were no surgical complications other than possible scar tissue formation on the penile shaft.

## Conclusion

Schwannomas of the penis are extremely rare and typically present as a solitary, asymptomatic, painless and slow-growing tumor. The rarity in this case is that our patient presented with painful nighttime erections. Based on the penile neuroanatomy, penile schwannomas at the mid shaft should originate from the dorsal nerve of the penis. Our hypothesis is that the schwannoma lies along the axis of the dorsal penile nerve and compression of this nerve occurs during his erection causing pain. However, there are limited presentations of painful erections in penile schwannomas, and we hope that future studies can help confirm this theory.

## Data Availability

Not applicable.

## Abbreviation

MRI: Magnetic resonance imaging
